# A Health Needs Assessment of the Filipino American Community in the Greater Las Vegas Area

**DOI:** 10.1089/heq.2018.0042

**Published:** 2018-11-27

**Authors:** Saruna Ghimire, Prescott Cheong, Lawrence Sagadraca, Lung-Chang Chien, Francisco S. Sy

**Affiliations:** Department of Environmental and Occupational Health, School of Community Health Sciences, University of Nevada, Las Vegas (UNLV), Las Vegas, Nevada.

**Keywords:** chronic conditions, dietary behaviors, Filipino American, health needs assessment, Las Vegas

## Abstract

**Purpose:** The number of Filipino Americans in Las Vegas, Nevada, is growing considerably, but no research to date has assessed the specific health needs of this burgeoning population. Thus, this study aims to assess health behaviors, perceived community health problems, and self-reported diseases/conditions among Filipino Americans in the Greater Las Vegas area and evaluate any difference by gender.

**Methods:** A cross-sectional survey was conducted among 200 Filipino American adults residing in the Greater Las Vegas area using a prevalidated instrument.

**Results:** The self-reported prevalence of hypertension, high cholesterol, and diabetes was 48%, 46%, and 25%, respectively. Adverse health behaviors, in terms of insufficient exercise and diets lacking in fruits and vegetables, were noted among our participants. Approximately 67% of participants reported exercising less than the recommended 150 min of physical activity per week and <3% of the study population ate the recommended five servings of fruits and vegetables a day. On the contrary, consumption of sweet snacks and salty condiments was high. More than two-thirds of respondents indicated that the Filipino American community should address the identified health conditions.

**Conclusions:** The high self-reported prevalence of hypertension, high cholesterol, and diabetes demonstrates a pressing public health problem among Filipino Americans in Las Vegas. Given that our study population comprised predominantly college-educated, middle-income, and insured individuals, the findings may be underestimated and thus the actual disease prevalence may be even higher. Results of this survey will be used to develop future interventions for the Filipino American community in Las Vegas using the principles of community-based participatory research.

## Introduction

With an estimated population of 3.4 million in the United States, Filipino Americans represent the third-largest ethnic minority group. It is the second largest Asian American subgroup after Chinese Americans.^1,2^ The Filipino ethnic group represented ∼19.7% of the total Asian American population of the United States in 2010.^3^ Moreover, Filipinos were the second most rapidly increasing Asian group in the United States after Asian Indians, with a 44% population growth rate from 2000 to 2010.^1^ The percent increase in the population of Filipino Americans was 28% from 1990 to 2000 and 44% from 2000 to 2010, respectively.^1,2^

In Las Vegas, Nevada, Filipino Americans represent the largest Asian American subpopulation with an estimated population of 108,141 in Census 2010.^1,4^ According to an analysis from the Pew Research Center, citing American Community Survey data from 2013 to 2015, Las Vegas was one of the top metropolitan cities where Filipino Americans lived.^5^ Between 2000 and 2010, the growth rate among Filipinos reached 158% in Las Vegas.^4^ In total, Filipino Americans represent almost 3.5% of the entire population of Las Vegas and over half of the Asian American population.^3,4^ Part of the reason that Las Vegas holds such a large Filipino population was due to recruitment of skilled health care workers from the Philippines, brought in to alleviate Nevada's nursing shortage and bolster the state's health care infrastructure.^6^ Additionally, it is also believed that affordable housing, Nevada's lower taxes, and the opportunity to work in casinos lured many ethnic groups, including Filipinos, to Las Vegas.^7^ Despite being one of the largest and rapidly expanding ethnic groups, health needs of Filipino Americans in Las Vegas are poorly understood and not fully assessed.^3^

Often times, health data on Filipino Americans are lacking because they are typically aggregated with other Asian American subgroups.^4^ In fact, several national surveys collect only a limited amount of subgroup information from Asian American respondents.^8^ Given the considerable social and economic diversity within the Asian American community, data aggregation seemingly assumes that Asian Americans are a homogeneous population.^4,9^ Consequently, this practice yields critical research implications as data aggregation masks the health needs of the most vulnerable groups in the Asian American community.^9^ Researchers and Asian American advocates have recognized this problem, and efforts to collect and report granular data for Asian Americans have increased over time.^8^

The limited number of available studies specifically focusing on Filipino American populations suggests that their overall health outcomes are worse compared with other racial groups and Asian American subgroups.^2,10,11^ A recent study examining hypertension among Filipino Americans in Greater Philadelphia reported rates as high as 67.5%.^10^ Another study from Las Vegas identified acculturation as a predictor of body–mass index (BMI), wait circumference, and waist–hip ratio among Filipino Americans with hypertension.^12^ Moreover, the 16.1% prevalence of type 2 diabetes among a Filipino American population sample in Houston, Texas, is significantly higher than the national average of 9.3%.^11^ Another study in California found that smoking prevalence was higher among Filipino Americans (24%), exceeding rates reported for non-Hispanic whites (19%).^13^ Alcohol consumption among Asian American adolescents revealed that Filipino Americans had the highest lifetime (29.3%) and past-month (10.3%) use.^14^ In addition, gender differences in certain health behaviors, especially smoking and drinking, among Filipino Americans have been reported by previous studies.^15–17^ These numerous adverse health outcomes documented within the Filipino American community provide evidence of the need to perform systematic culturally sensitive studies that evaluate the overall health status of this population. Fortunately, health needs assessments have been previously conducted among Filipino Americans in California, New York, and Philadelphia.^10,18,19^

Considerable variation in health status among Filipino Americans has been attributed to the rich cultural diversity in terms of language, place of origin, cultural heritage, acculturation, and other sociodemographic indicators.^20–22^ Immigrant status, acculturation, and length of stay in the United States have been linked to obesity and poor health outcomes among Filipino Americans.^20,21^ Variations in overweight and obesity status have also been correlated with the individual's island of origin in the Philippines.^20,21^ Filipinos who originated from the central region in the Philippines, compared with other regions, are more likely to be overweight, which could be potentially explained by regional variation in degree of urbanization, culture, diet, and physical activity patterns in the Philippines.^20^ Based on the geography and culture, different islands in the Philippines show diversity in terms of behaviors related to diet and/or physical activity, which may predispose people from one island group more toward obesity than another.^22^ Furthermore, the complex cultural diversity among the Philippine island regions has created considerable heterogeneity in behaviors related to diet and/or activity.^22^ Filipino Americans in Houston, Texas, had an estimated 16.1% overall prevalence of type 2 diabetes, and the region of birth was one of the significant risk factors.^11^ A previous study showed that residence in New Jersey, compared with the New York City boroughs, was associated with a higher risk of being overweight, suggesting variation in health outcomes based on the area of residence in the United States.^23^ Therefore, the health status and corresponding health needs of Filipinos residing in Las Vegas are unclear.

Food insecurity within the Filipino community is an understudied topic. Limited available studies have indicated that US-born Asians had similar food insecurity rates to US-born whites.^24^ Again, significant heterogeneity in the food insecurity prevalence among students attending the University of Hawaii was noted within the Asian American subgroups, with Filipino American students reporting the highest rates of food insecurity at 33%.^25^

Two current political issues, President Trump's travel ban for new immigrants and the proposed repeal of the Affordable Care Act (ACA), may have a direct impact on mental health and access to health care. Since foreign-born workers make up nearly a quarter of the health care workforce and many of these workers come from China, India, and the Philippines, such recent political events may also have an impact on the US health care workforce.^26^ These current events may have impacted the Filipino community because of their immigration history and minority status. Thus, it was timely and important to capture any political events that could potentially impact our study participants' mental health.

Consequently, this study aimed to assess health behaviors, perceived community health problems, and self-reported diseases/conditions among Filipino Americans in the Las Vegas area and evaluate any difference by gender. Additionally, we also aimed to assess prevalence of food insecurity and anxiety about President Trump's travel ban and the proposed repeal of ACA.

## Methods

### Study procedure

A total of 200 (*n*=200) Filipino American adults residing in the Greater Las Vegas area were recruited to participate in this study, which was conducted from April 2017 to August 2017. The Greater Las Vegas area includes the cities of Las Vegas, North Las Vegas, Henderson, Summerlin, Paradise, Spring Valley, and Enterprise.

The sample size was calculated based on a statistical formula, sample size (ss)=z^2^pq/d^2^, taking z=1.96 at 95%, p (prevalence)=50%, q=1 – p, and d (allowable error)=7%. Furthermore, sample size for finite population was calculated by using the formula, *n*=ss/(1 + (ss −1)/p), and finite population size (p) of 39,303 (total population of Filipino Americans residing in the Greater Las Vegas area^3^). Thus, the population-based sample size at 95% confidence interval (CI) and 50% prevalence was 195, which was rounded to 200.

Before the data collection process, the research team identified and reached out to numerous Filipino American community leaders in Las Vegas to inquire about opportunities and sites where surveys could be distributed and completed. Study participants were recruited through a convenient sampling approach from picnics, churches, and social events organized by Filipino community-based organizations in Las Vegas. Participants were also recruited at a popular Filipino grocery store. The survey was available in paper form to be completed at data collection sites. An online version was also available through SurveyMonkey^™^, which was distributed during our recruitment events. The eligibility criteria included (1) self-identifying as Filipino, (2) residing in the Greater Las Vegas area, and (3) being aged 18 years and over. Participants were recruited until the desired sample size was achieved. A total of 238 survey questionnaires were received, but 38 were discarded due to incompleteness.

### Ethics and consent

This study received approval from the Institutional Review Board at University of Nevada, Las Vegas (Protocol No. 1046862-2). The self-administered instrument packet contained an informed consent form, which provided a detailed explanation of the study aims as well as the procedures to be followed. To maintain anonymity of our survey participants, we did not collect any personally identifiable information. Participants were also informed of the voluntary nature of completing our survey and were subsequently requested to provide verbal consent.

### Data collection and variables

The research team included a principal investigator and project team members who were University of Nevada, Las Vegas, graduate students in the School of Community Health Sciences. The research team members were involved in every phase of study planning and were acquainted with the research objectives, study tools, sampling strategy, and data collection techniques. Data were collected through self-administered questionnaires. Upon arrival at data collection sites, local community leaders were asked to help facilitate distribution and completion of the study. Information about the study's purpose and requirements, informed consent process, and voluntary nature of this survey was explained to Filipino community leaders who assisted in data collection before they approached prospective participants.

A previously validated instrument was used for this study.^10^ Details of the Filipino health needs survey instrument were discussed in a previous study.^10^ Briefly, the tool included questions on demographics, basic acculturative traits, health behaviors, health conditions, and perceived community health issues. For our study in Las Vegas, new questions were added that centered on BMI, women's health, food insecurity, and stress and anxiety related to the President's travel ban and possible repeal of the ACA.

#### Demographics

Demographic information on participant's age, gender, marital status, education, employment, employment type, family's annual income, family type, height, and weight was collected by self-report. Except for age, height, and weight, all other variables were categorical and responses are provided in Table 1. Family type was classified as nuclear (parents with children under the age of 18), joint (parents with children above the age of 18), and extended family (family, including siblings above 18, aunts, uncles, and cousins, all living in the same household). Self-reported height and weight of participants were converted into BMI in kg/m^2^. Participant's weight status was characterized as underweight (BMI <18.5), normal weight (18.5 ≤ BMI <23), overweight (23 ≤ BMI <27.5), and obese (BMI ≥27.5) as per the World Health Organization (WHO) recommendation for Asians.^27^

**Table 1.** **Demographic Characteristics of Participants: Filipino American Health Survey in the Greater Las Vegas Area**

Demographics (*N*=200)	*n*	%
Age, years, mean±SD (missing=6)	49.4±18.1	—
Gender		
Male	70	35.0
Female	130	65.0
Missing	0	0.0
Marital status		
Married/living as married	112	56.0
Never married	46	23.0
Divorced/separated/widowed	39	19.5
Missing	3	1.5
Educational status		
High school or below	36	18.0
College or associate	109	54.5
Graduate and above	52	26.0
Missing	3	1.5
Employment status		
Employed	123	61.5
Unemployed	21	10.5
Retired	54	27.0
Missing	2	1.0
Current employment^a^ (*N*=123)		
Health care worker	42	34.1
Administration or other professional	16	13.0
School employee	15	12.2
Casino employee	14	11.4
Grocery, retail, and food	12	9.8
Self-employed	12	9.8
Government	4	3.3
Other	7	5.7
Missing	1	0.8
Annual household income		
Less than $20,000	40	20.0
$20,000–$40,000	48	24.0
Above $40,000	102	51.0
Missing	10	5.0
Family type		
Nuclear	87	43.5
Joint	72	36.0
Extended	27	13.5
Missing	14	7.0
BMI, kg/m^2^, mean±SD (missing=7)	25.3±6.6	—
Weight status		
Underweight	12	6.0
Normal weight	59	29.5
Overweight	74	37.0
Obese	48	24.0
Missing	7	3.5

^a^ Only asked to participants who were currently employed.

BMI, body–mass index; SD, standard deviation.

#### Acculturative traits

The acculturative traits assessed were place of birth, number of years lived in the United States, reading and spoken English proficiency, language spoken at home, and food type usually eaten (Western, Filipino, or both). Participants were asked closed-ended questions with response options, as reported in Table 2.

**Table 2.** **Acculturation Characteristics of Study Participants: Filipino American Health Survey in the Greater Las Vegas Area**

Acculturation characteristics (*N*=200)	*n*	%
Born in the United States		
Yes	36	18.0
No	162	81.0
Missing	2	1.0
Years living in the United States, mean±SD (*N*=162; missing=14)	24.9±15.1	—
Reading English proficiency		
Well	34	17.0
Very well	163	81.5
Missing	3	1.5
Spoken English proficiency		
Not well	7	3.5
I speak English well	62	31.0
I speak English fluently	129	64.5
Missing	2	1.0
Language spoken at home		
English	73	36.5
Filipino	64	32.0
Both	59	29.5
Missing	4	2.0
Food type usually eaten		
American or Western food	18	9.0
Filipino food	42	21.0
Both	135	67.5
Missing	5	2.5

#### Health behaviors

Participants' dietary consumption patterns (fruits, vegetables, meat, fish, salt, and sugar), alcohol, smoking, tobacco use, and physical activity were asked. The number of daily servings of meat and fish consumption in a typical week and the weekly consumption of sweets and fast foods were assessed. Frequency (never, often, and every meal) of the addition of salt and salty condiments/sauces to foods was asked. For physical activity, participants were asked to report the minutes of activities performed each day and the number of days such activity was performed in a typical week. The minutes of physical activity per week were then categorized into physically inactive (<150 min) and active (≥150 min) based on the Physical Activity Guidelines for Americans.^28^ Participants were asked to report the average servings of fruits and vegetables in a typical week, which was then categorized into less than five servings and five servings or more per day, as suggested by the Dietary Guidelines for Americans.^29^ To assess smoking, participants were asked “How often do you smoke cigarettes?” and they could select one of the following options: every day, some days, and not at all. To assess indoor smoking exposure, participants were asked about the number of days in a week someone, other than the participants, smoked inside the home. It was then recoded as never if no one smoked indoor, else it was recoded as some days. Participants were asked if they currently used other tobacco products (e.g.: chewing tobacco, inhaled tobacco, or placed tobacco on the inside of lips) every day, some days, or not at all. The responses, every day and some days, were recoded as yes, and not at all was recoded as no. Alcohol consumption was assessed by two items: number of days the participant had at least one drink of any alcoholic beverage in the past month and the number of drinks they had on the days they drank. It was then recoded into a dichotomized yes/no response during data analysis; recoded into yes if the participant reported having at least one alcoholic drink, else it was recoded as no.

#### Health conditions

To capture self-reported health status, participants were asked a number of multiple-choice and multiple-response questions that requested information on all the listed health conditions (high blood pressure, high blood cholesterol, heart attack, angina or coronary heart disease, stroke, asthma, chronic obstructive pulmonary disorder, arthritis, depressive disorder, kidney disease, diabetes, oral cancer, breast cancer, hepatitis B, and any other chronic conditions) that they were diagnosed with by a doctor, nurse, or other health care professional. Participants were also asked if they were currently taking medication for their reported disease(s).

#### Other health issues

Participant's access to health care was assessed in terms of having a doctor for a regular health checkup, time since last general health checkup, type of health insurance, language spoken with a physician, and getting time off from work for doctor appointments. Participants were also asked about other health issues, such as ever being tested for HIV, vaccinated against hepatitis B, checked blood cholesterol, time since last dental cleaning, and any tooth extractions.

#### Perceived community health issues

Participants' perceptions of a major health problem in the Filipino American community and those related to the Filipino diet and genetics were asked using multiple-response questions. Finally, participants were asked about major health conditions that needed to be better addressed in the Filipino American community and the stakeholder responsible for addressing the problems, both using multiple-response questions. The different questions asked to assess perceived community health issues and the corresponding responses provided within each question are shown in Figure 2.

#### Men's and women's health

Men were asked about the time since their last prostate-specific antigen (PSA) test. Likewise, women were asked about the time since their last mammogram, clinical breast examination, and Pap smear. In a follow-up question, the reason for undertaking the mammogram or the barriers preventing the mammogram was asked, respectively, to the females who had had a mammogram in the past and those who did not. Additionally, females were also asked about the frequency of the breast self-examination and their preference for a female physician or health professional for a general checkup.

#### Food insecurity

To assess participants' financially based food insecurity status, the previously validated and widely used six-item short form of the Food Security Survey Module was utilized.^30^ These series of questions asked participants about the affordability of food eaten or needed in the household in the last 12 months. Each item in the food security scale was reduced to the category of affirmative or negative, as per the recommendation.^31^ The sum of affirmative responses to the six questions in the module is the household's raw score on the scale. The food security status was assigned as high (raw score 0–1), low (raw score 2–4), and very low (raw score 5–6).^31^

#### Anxiety related to the travel ban and possible ACA repeal

Participants were asked if the recent executive order of President Trump on a travel ban for new immigrants and the possible repeal of the ACA had any impact on them (yes/no/don't know) and about the personal level of anxiety/stress surrounding the two issues (none, a little stressed, moderately stressed, and extremely stressed). Furthermore, in multiple-choice questions (responses listed in Table 6), participants were asked to choose the source of anxiety/stress surrounding these two issues.

### Data processing and statistical analyses

Data management and analyses were completed using IBM SPSS22 for Windows (SPSS, Inc., Chicago IL). To ensure the accuracy and quality of data entry, the entered data were thoroughly rechecked for correctness. Descriptive statistics for all variables are provided in this study. Numerical variables are expressed as mean and standard deviation and categorical variables as frequency and percentage. Comparisons of means between the genders were made by an independent *t*-test, while frequency distribution was evaluated by Pearson's chi-square test. A *p*-value of <0.05 was considered statistically significant in this study.

## Results

### Demographics

A total of 200 participants, 70 men and 130 women, participated in this survey (Table 1). The mean age of participants was 49 years and ranged between 18 and 85 years. Majority of the study participants were married (56.0%), college (54.5%) or university (26.0%) graduates, and employed (61.5%) at the time of the survey. Most respondents were employed as health care workers (34.1%). About half of the participants lived in a nuclear family (43.5%) and in a family with an annual household income greater than $40,000 (51.0%). The mean BMI of participants was 25.3 kg/m^2^, and a high proportion of participants were overweight (37.0%) or obese (24.0%) (Table 1). In the bivariate analysis, except for marital status, there was no significant difference in other demographic characteristics between male and female participants (data not shown).

### Acculturative traits

Eighty-one percent of study participants were born outside the United States (85.8% of those born outside the United States were born in the Philippines). However, many respondents had spent a large portion of their lives in the United States; the mean years of US residence was 25 years (Table 2). Majority of participants reported being highly proficient in reading and speaking English. Furthermore, the majority of participants spoke English at home (36.5%) and usually ate both American and Filipino food (67.5%) (Table 2). Again, participants did not significantly differ in any of the measured acculturative traits by gender (data not shown).

### Health behaviors

The self-reported dietary consumption revealed that participants exhibited poor dietary practices. On the one hand, consumption of fruits and vegetables was low, while on the other hand, consumption of sweets, salty condiments/sauces, and fast food was high (Table 3). Less than 3% of our study population consumed recommended daily servings of five fruits and vegetables. Approximately 37.6% of respondents consumed five or more servings of sweets a week, and 69.4% of respondents stated that they added salty condiments to their meals often or with every meal. Most of the participants neither smoked (87.4%) or used tobacco (94.3%) nor were they exposed to indoor smoking at home (81.6%). Smoking and tobacco consumption were significantly higher among males compared with females. Alcohol was consumed by 42% of participants; a significantly higher proportion of males than females consumed alcohol. Physical activity among participants was low, and only 32.6% of participants met the recommended exercise dose of 150 min or more per week (Table 3).

**Table 3.** **Health Behaviors of Study Participants: Filipino American Health Survey in the Greater Las Vegas Area**

Health behaviors	Total (*N*=200) *n* (%)^a^	Male (*N*=70) *n* (%)^a^	Female (*N*=130) *n* (%)^a^	*p*^b^
Fruit and vegetable servings (missing=16)				
<5 Servings/day	179 (97.3)	64 (97.0)	115 (97.5)	0.845
≥5 Servings/day	5 (2.7)	2 (3.0)	3 (2.5)	
Fish servings (missing=3)				
<2 Servings/day	122 (61.9)	45 (65.2)	77 (60.2)	0.485
≥2 Servings/day	75 (38.1)	24 (34.8)	51 (39.8)	
Meat servings (missing=3)				
<2 Servings/day	67 (34.0)	16 (23.2)	51 (39.8)	**0.02**
≥2 Servings/day	130 (66.0)	53 (76.8)	77 (60.2)	
Sweet servings (missing=3)				
<5 Servings/day	123 (62.4)	46 (66.7)	77 (60.2)	0.368
≥5 Servings/day	74 (37.6)	23 (33.3)	51 (39.8)	
Salt addition to food (missing=2)				
Never	57 (28.8)	17 (24.3)	40 (31.3)	0.11
Often	105 (53.0)	44 (62.9)	61 (47.7)	
Every meal	36 (18.2)	9 (12.9)	27 (21.1)	
Adding salty condiments/sauces to food (missing=1)				
Never	61 (30.7)	21 (30.0)	40 (31.0)	0.387
Often	116 (58.3)	44 (62.9)	72 (55.8)	
Every meal	22 (11.1)	5 (7.1)	17 (13.2)	
Fast food consumption (missing=1)				
Never	33 (16.6)	14 (20.0)	19 (14.7)	0.542
Once per week	94 (47.2)	30 (42.9)	64 (49.6)	
More than once per week	72 (36.2)	26 (37.1)	46 (35.7)	
Current smoking status (missing=1)				
Every day	13 (6.5)	8 (11.6)	5 (3.8)	**<0.001**
Some days	12 (6.0)	10 (14.5)	2 (1.5)	
Not at all	174 (87.4)	51 (73.9)	123 (94.6)	
Indoor smoking exposure (missing=48)				
Never	124 (81.6)	40 (78.4)	84 (83.2)	0.477
Some days	28 (18.4)	11 (21.6)	17 (16.8)	
Tobacco consumption (missing=7)				
No	182 (94.3)	59 (88.1)	123 (97.6)	**0.006**
Yes	11 (5.7)	8 (11.9)	3 (2.4)	
Alcohol consumption (missing=17)				
No	106 (57.9)	26 (39.4)	80 (68.4)	**<0.001**
Yes	77 (42.1)	40 (60.6)	37 (31.6)	
Minutes of physical activity per week (missing=18)				
<150 Min	124 (67.4)	42 (61.8)	82 (70.7)	0.213
≥150 Min	60 (32.6)	26 (38.2)	34 (29.3)	

Significant *p*-values are bolded.

^a^ Low percentage based on nonmissing data in total and by gender.

^a^ *p*-Values are from an independent samples *t*-test, all others are from a chi-square test.

### Health conditions and other health issues

**FIG. 1.**
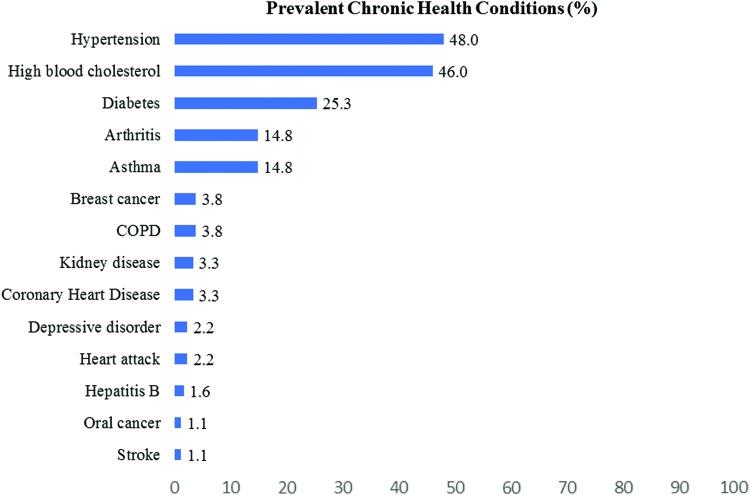
Health conditions among the study participants: Filipino American health survey in the Greater Las Vegas area. All values are multiple-response percentages.

Hypertension (48.0%), high cholesterol (46.0%), and diabetes (25.3%) were the most prevalent conditions among the participants (Fig. 1). Most of the participants had a doctor (76.9%) and health insurance (89.0%) for a regular health checkup. For the majority of participants, no barriers to health access were observed in terms of language spoken with physician and getting time off from work for health appointments (Table 4).

**Table 4.** **Health Issues Among Study Participants: Filipino American Health Survey in the Greater Las Vegas Area**

	*n*	*%*
Has a doctor for regular health checkup		
Yes	140	70.0
No	42	21.0
Missing	18	9.0
Time since last general health checkup		
Within the past year	145	72.5
More than a year	36	18.0
Missing	19	9.5
Type of health insurance		
No insurance	14	7.0
Medicare	46	23.0
Medicaid	13	6.5
School/employer-sponsored	92	46.0
Government subsidized/military	11	5.5
Other	10	5.0
Not sure	8	4.0
Missing	6	3.0
Language spoken with physician		
English	143	71.5
Filipino	13	6.5
Both	34	17.0
Do not have a physician	4	2.0
Missing	6	3.0
Time off from work for doctor's appointments		
No	140	70.0
Yes	20	10.0
I do not work, so this is not an issue	33	16.5
Missing	7	3.5
Ever tested for HIV		
Yes	41	20.5
No/not sure	141	70.5
Missing	18	9.0
Ever had hepatitis B vaccination		
Yes	101	50.5
No	77	38.5
Missing	22	11.0
Has blood cholesterol been checked?		
Yes	141	70.5
No/not sure	58	29.0
Missing	1	0.5
Time since last blood cholesterol checkup		
Within the past year	133	66.5
More than a year	32	16.0
Never	33	16.5
Missing	2	1.0
Time since last dental cleaning by a dentist		
Within the past year	138	69.0
Within the past 2 years	26	13.0
Within the past 5 or more years	25	12.5
Never	9	4.5
Missing	2	1.0
Ever underwent a tooth extraction in Philippines		
Yes	105	52.5
No/not sure	85	42.5
Missing	10	5.0

### Perceived community health issues

**FIG. 2.**
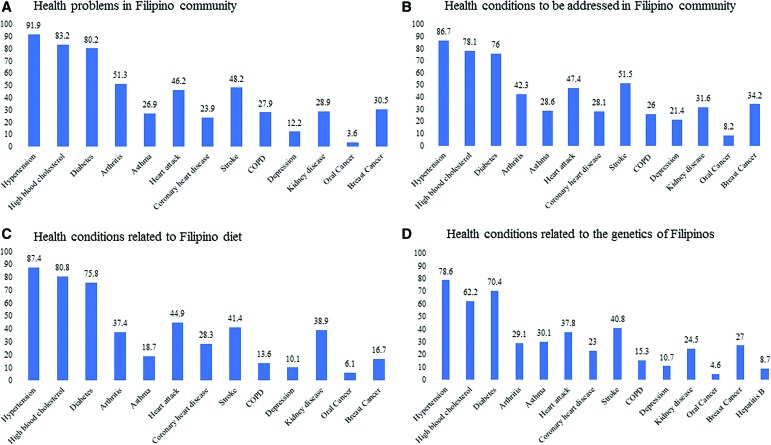
**(A-D)** Perceived community health issues among the study participants: Filipino American health survey in the Greater Las Vegas Area. All values are multiple-response percentages.

Our study participants perceived high blood pressure (91.9%), high blood cholesterol (83.2%), diabetes (80.2%), and arthritis (51.3%) as major health problems among adults in the Filipino American community that should be better addressed in the Filipino American community among adults (Fig. 2). The participants also believed that these health conditions were related to the Filipino diet and genetics (Fig. 2). Most of the participants stated that it was the responsibility of the Filipino American community (73.5%), local doctors and health care professionals (68.3%), and Filipino American doctors and health care professionals who can speak the native language (55.6%) to address health conditions affecting their community. A lesser proportion of participants identified that existing health problems in the Filipino American community should be addressed by the local health department (43.9%), state health department (48.7%), federal government (35.4%), and pharmaceutical companies (26.5%).

### Men's and women's health

Among women aged 40 and above, ∼23% had never had a mammogram or not had one in the past 5 years (Table 5). Among those who had had a mammogram, it was a part of a routine checkup. Likewise, 23% of the women, aged 40 and above, had never had a clinical breast examination or not had one in the past 5 years, and 43% had never had a Pap smear or not had one in the past 5 years. About three-fourths of the women preferred a female physician or health professional. Among men, aged 40 and above, 27% had never had a PSA test (Table 5).

**Table 5.** **Men's and Women's Health Among Study Participants: Filipino American Health Survey in the Greater Las Vegas Area**

	*n*	%
Women's health (age ≥40, *n*=86)		
Time since last mammogram		
Within the past year	47	54.7
Within the past 2 years	18	20.9
Within the past 5 or more years	16	18.6
Never	4	4.7
Missing	1	1.2
Reason for taking mammogram		
Part of a routine checkup	73	90.1
Because of a breast problem	2	2.5
Because you had previous breast cancer	3	3.7
Missing	3	3.7
Reason for not taking a recommended mammogram		
Lack of insurance/could not afford	12	57.1
I don't have time	9	42.9
Time since last clinical breast examination		
Within the past year	41	47.7
Within the past 2 years	19	22.1
Within the past 5 or more years	13	15.1
Never	7	8.1
Missing	6	7.0
Frequency of a breast self-examination		
Once a week	17	19.8
Once a month	27	31.4
Once a year	21	24.4
Once within the past 2 years	7	8.1
Never done a breast self-examination	11	12.8
Missing	3	3.5
Time since last Pap smear		
Within the past year	31	36.0
Within the past 2 years	16	18.6
Within the past 5 or more years	34	39.5
Never	3	3.5
Missing	2	2.3
Preference of a female physician or health professional (all females, *n*=130)		
Yes	96	73.8
No/no preference	28	21.5
Missing	6	4.6
Men's health (age ≥40, *n*=45)		
Time since last PSA test		
Within the past year	20	44.4
Within the past 2 years	5	11.1
Within the past 5 or more years	5	11.1
Never	12	26.7
Missing	3	6.7

PSA, prostate-specific antigen.

### Food insecurity

Approximately 73.2% of participant households were categorized as high/marginal food security status; 21.6% reported low food security status, and 5.3% reported very low food security status.

### Anxiety related to the travel ban and possible ACA repeal

Only 9.5% of participants were impacted by the recent executive orders related to the travel ban of new immigrants, but about half of the participants were stressed to some level by it (Table 6). Participants were worried that their friends and family back in the Philippines would not be able to come to the United States (43.9%). Likewise, about a quarter of the participants were impacted by the possible repeal of the ACA, and around 35% of participants were stressed to some level by it. Participants were worried that a possible repeal of ACA would affect the general Filipino American community (36.8%) and participants' (16.8%) or their families' (20.0%) ability to keep their health insurance (Table 6).

**Table 6.** **Anxiety Related to the Travel Ban and Possible Affordable Care Act Repeal: Filipino American Health Survey in the Greater Las Vegas Area**

	*n*	%
Impacted by the recent executive orders related to the travel ban		
Yes	19	9.5
No	153	76.5
I don't know	22	11.0
Missing	6	3.0
Personal level of anxiety/stress surrounding the executive orders		
Not stressed	102	51.0
A little stressed	56	28.0
Moderately stressed	23	11.5
Extremely stressed	5	2.5
Missing	14	7.0
Reason for anxiety/stress for the executive orders related to the travel ban (multiple responses)		
Worried about own future	17	11.0
Worried about family's future	17	11.0
Worried about friends and family back in the Philippines	68	43.9
No affect	61	39.4
Other impacts	12	7.7
Any impact of the possible repeal of the Affordable Care Act		
Yes	48	24.0
No	98	49.0
I don't know	43	21.5
Missing	11	5.5
Personal level of anxiety/stress surrounding a possible repeal of the Affordable Care Act		
Not stressed	110	55.0
A little stressed	39	19.5
Moderately stressed	21	10.5
Extremely stressed	10	5.0
Missing	20	10.0
Source of anxiety/stress for the possible repeal of Obamacare (multiple responses)		
Worried about own ability to keep health insurance	26	16.8
Worried about family's ability to keep health insurance	31	20.0
Worried it would affect the Filipino American community	57	36.8
No effect	58	37.4
Other impacts	7	4.5

## Discussions

The main purpose of our study was to determine health behaviors, perceived community health problems, and self-reported diseases/conditions among Filipino Americans in the Greater Las Vegas area. In this study, we found that Filipino Americans in the Greater Las Vegas area are highly acculturated and exhibited poor health behaviors in terms of diet and exercise. Hypertension, high cholesterol, and diabetes were the most prevalent self-reported chronic conditions among the participants. Interestingly, the majority of respondents correctly identified hypertension, high cholesterol, and diabetes as major health issues affecting the Filipino American community in Las Vegas. Study participants also indicated that the responsibility of addressing these particular chronic diseases/conditions largely falls upon the Filipino American community and local Filipino American health providers and, to some extent, local and state health departments. These results suggest that a community-based participatory research (CBPR) framework may be an appropriate and effective approach to meeting the health needs of Filipino Americans in the Greater Las Vegas area.

According to the National Institute on Minority Health and Health Disparities, CBPR employs an active, equal, and collaborative partnership between scientific researchers and community stakeholders to collectively address the targeted health issue.^32^ Key to this relationship is the participation of community members throughout each facet of the project at hand, which includes planning, research, program design, implementation, and evaluation.^32^ CBPR principles have been successfully utilized to address health problems within the Asian American community and Filipino American community in New York and New Jersey.^33–35^ For example, a culturally tailored community health worker intervention for hypertensive Filipino Americans revealed that strong academic–community partnerships, guided by a CBPR framework, were essential for ultimately creating a culturally acceptable, feasible, and efficacious intervention.^34^ Future research aimed at improving the health status of Filipino Americans in Las Vegas should apply the principles of CBPR to properly address social, cultural, and health needs of this unique population.

In general, Filipino Americans are better educated, more likely to have professional jobs, and have higher incomes, which we also found in our study in Las Vegas.^36,37^ Acculturation is a process of culture learning and behavioral adaptation as a result of contact with culturally dissimilar people or groups, with subsequent changes in the original cultural patterns, and is very common among immigrant populations in the United States.^38^ Findings from this study and others suggest that Filipino Americans are highly acculturated.^10,39^ Although more than 80% of participants in our study were immigrants, many reported living in the United States for large portions of their lives and were fluent in speaking and reading English, findings similar to a previous study from Philadelphia.^10^ Acculturation strongly impacts immigrant health status.^40^ The healthy immigrant hypothesis states that immigrants, in general, are healthier than their corresponding racial/ethnic counterparts born in the host country. However, these health advantages decline with years of residence in the host country.^41,42^ Among Filipino Americans, acculturation provides socioeconomic benefits, but may have both positive and negative consequences within a health context.^39^ Socioeconomic improvement as a positive result of immigration and acculturation provides Filipino Americans with an opportunity to earn a higher income, obtain higher education, greater access to health care, and better health outcomes through prevention and early diagnosis and treatment of diseases.^43^

This study observed a high proportion of participants with overweight or obese status, which is similar to other studies.^20,44^ Filipino American adults are 70% more likely to be obese compared with the overall Asian population.^44^ Lack of physical activity combined with poor dietary practices may explain the disproportional overweight/obesity among Filipino Americans. The Physical Activity Guidelines for Americans recommend at least 150 min of physical activity per week.^28^ Similarly, the Dietary Guidelines for Americans recommend consuming at least five servings of fruits and vegetables daily.^29^ On the contrary, only 32.6% and 2.7% of our study participants met the recommended levels of physical activity and daily servings of fruits and vegetables, respectively. Given that most of our participants were educated and lived in a food secure household with an annual household income above $40,000, food insecurity was unlikely to have played a role in limited consumption of healthy foods. Low physical activity level among our participants and the general Filipino American community is primarily attributed to lack of time among Filipino Americans.^10,11,19,45^ Moreover, the high consumption of sweets, salty condiments/sauces, and fast food among Filipino Americans may contribute to higher body mass.^10,19,46,47^ Furthermore, the poor dietary behavior as well as high rates of overweight status/obesity may also be explained by acculturation. In a previous study, acculturation to the United States over time was associated with unhealthy food choices causing shifts from traditional diets of vegetables, fish, and whole grains to the more processed, high-fat, and sugary foods that are popular and available to immigrants, which can lead to obesity.^48^ Immigrant status, acculturation, and length of stay in the United States have been linked to obesity among Filipino Americans.^20^ In particular, it is culturally acceptable in a Filipino American community for children to be overweight.^45^

Our findings of the high prevalence of hypertension, dyslipidemia, and type 2 diabetes are supported by earlier studies.^10,11,49–52^ Nonobese Filipino Americans had more than twice the odds of diabetes compared with non-Hispanic whites, even after correcting for several known risk factors.^53^ Acculturation, poor dietary practice, and lack of physical activity, which were common among our participants, have been implicated in a variety of chronic diseases among Filipino Americans.^54,55^ Unhealthy diet and physical inactivity are important modifiable risk factors for hypertension, dyslipidemia, and type 2 diabetes.^56–59^ Physical inactivity was one of the determinants of type 2 diabetes among Filipino Americans in Texas.^11^ Additionally, diet combined with physical exercise has been identified as the most effective preventive strategy in reducing the incidence of diabetes.^60^ The role of salt and salty condiments in hypertension is well established.^61^ High salt intake is an important behavioral and biological risk factor for hypertension.^62^ Since a modest reduction in salt intake can effectively lower blood pressure, the role of dietary sodium reduction is promising and is considered as one of the most cost-effective and easily implemented strategies in dietary approaches to stop hypertension.^57,61^

Alcohol consumption was also high among the participants, which is supported by previous studies.^14,15,63^ Coincidentally, all these risk factors were high among our study participants and thus may explain the high prevalence of chronic conditions. Gender-specific smoking and drinking differences were noted, which is supported by previous literature.^15–17^ The findings may be explained by the fact that in Filipino culture, smoking and drinking by men, but not women, are socially acceptable. This was further supported by the fact that acculturated adult females had a higher smoking rate than the less acculturated.^17^

Breast cancer survival disparities exist for Filipinas in Nevada.^64^ A study among women residing in southern Nevada found that Filipinas had a higher risk of death than white women, primarily attributable to the stage at diagnosis.^64^ Despite having a better socioeconomic advantage, late stages of diagnosis among Filipinas accounted for their relative survival disadvantage in Nevada, which hints toward low intake of mammogram test as evident in our study; about 43% of our participants had either never had a mammogram or it has been more than 5 years since the last mammogram.^36,64^

Prostate cancer is the most common cancer in American men after skin cancer.^65^ Multiple studies have identified prostate cancer as a leading cancer in Filipino American men.^66–68^ Using data from the Surveillance, Epidemiology, and End Results (SEER) Program, the age-adjusted prostate cancer incidence rate for Filipino American men was 121.9 (95% CI: 117.3–126.6).^66^ A study among five Asian American ethnic groups (Chinese, Filipino, Vietnamese, Korean, and Japanese) in California found that Filipino American men had the highest incidence and mortality rates for prostate cancer.^68^ Given that early detection can reduce death rates among men with prostate cancer, the American Cancer Society recommends a yearly PSA test for men over the age of 50.^65^ On the contrary, about one-third of our participants never completed a PSA test. Furthermore, Filipino American men are more likely to be diagnosed with advanced-stage prostate carcinomas compared with other Asians and non-Hispanic whites,^67^ which provides a likely explanation for worse survival rates among Filipino American men and suggests poor utilization of screening measures. A study among Filipino American men in Hawaii found lack of awareness of the need for screening, reticence to seek health care when feeling well, fear of cancer diagnosis, financial issues, time constraints, and embarrassment as barriers to prostate cancer screening,^69^ whereas the presence of urinary symptoms, personal experience with family or friend who had cancer, and receiving recommendations from a health care provider regarding screening were facilitators for screening.^69^ The study recommended culturally relevant interventions to address barriers to prostate cancer screening participation and misconceptions about causes of prostate cancer.^69^

The findings of our study have many implications for Filipino American community advocates and stakeholders in the Greater Las Vegas area. In particular, local health care providers should be cognizant of the low cancer screening rates among Filipino Americans and recommend prostate cancer (male) and breast cancer screenings (female) for their Filipino American patients. On a public health level, local Filipino American community-based organizations and university researchers should work in unison to implement evidence-based interventions aimed at improving physical activity levels and diet among the Filipino community in an effort to decrease the prevalence and effects of hypertension, high cholesterol, and diabetes. These interventions should utilize a CBPR framework and also employ Filipino American facilitators to maintain linguistic and cultural appropriateness.

### Strengths, limitations, and generalizability

This is the first health needs assessment study in the Filipino American community in the Greater Las Vegas area. It provides a fuller health profile of Las Vegas' fastest growing and most diverse ethnic groups. Our findings may be limited in terms of the self-reported nature of the data. Often, self-reported data on positive health behaviors are overestimated, which may be more prominent in our study since a majority of our participants were educated and employed as health professionals and with prior knowledge of the disease and/or risk factor they may have under-reported or over-reported certain health behaviors.^70^ Nevertheless, self-report is one of the most feasible and cost-effective methods known for collecting data and can provide actionable information despite its limitations.^71^

Small sample size is a limitation. We followed a convenience sampling approach to recruit participants. Thus, the generalizability of study findings may be limited and may not represent the entire Filipino American population in the Las Vegas metropolitan area. Our findings may also be limited in terms of generalizability given that majority of the respondents were middle-aged, college educated, fluent in English, possessed incomes higher than $40,000, and insured. Our participants' characteristics favor a better health outcome and hint that the findings may be different, specifically worse health outcomes and greater health needs among Filipino Americans with lower education and income and lack of insurance or fluency in English communication—traits associated with health disparities and accessibility.

## Conclusions

Our study among Filipino Americans in Las Vegas, Nevada's vital and expanding minority group, is a first step toward identifying this community's health needs. The high self-reported rates of hypertension, high cholesterol, and diabetes demonstrate a pressing public health problem among Filipino Americans in Las Vegas. Given that our study population comprised predominantly college-educated, middle-income, and insured individuals, traits favoring positive health outcomes, our findings may be underestimated, and thus the actual burden may be even more alarming. More than two-thirds of respondents indicated that Filipino Americans should develop preventive interventions in their own communities, which demand an intervention based on a CBPR approach to address the high burden of the selected disease, that is, hypertension, high cholesterol, and diabetes. Thus, results of this survey will be used to develop future interventions for Filipino American communities in Las Vegas using the principles of CBPR. Findings will also help local stakeholders to understand the wider needs of the community, which is important in planning and provision of local health services, and to better serve the community.

## Abbreviations Used

ACA: Affordable Care Act
BMI: body–mass index
CBPR: community-based participatory research
CI: confidence interval
PSA: prostate-specific antigen
